# Negative pain management index scores do not necessarily indicate inadequate pain management: a cross-sectional study

**DOI:** 10.1186/s12904-018-0355-8

**Published:** 2018-08-24

**Authors:** Naoki Sakakibara, Takahiro Higashi, Itsuku Yamashita, Tetsusuke Yoshimoto, Motohiro Matoba

**Affiliations:** 1 Division of Health Services Research, Center for Cancer Control and Information Services, National Cancer Center, 5-1-1 Tsukiji, Chuo-ku, Tokyo, 104-0045 Japan; 2 Department of Palliative Care, St. Luke’s International Hospital, 9-1 Akashicho, Chuo-ku, Tokyo, 104-8560 Japan; 3 Department of Palliative Care, Aomori Prefectural Central Hospital, 2-1-1 Higashitsukurimichi, Aomori City, Aomori 030-8553 Japan; 4 Department of Palliative and Supportive Care, Chukyo Hospital, 1-1-10 Sanjo, Minami-ku, Nagoya City, Aichi 457-8510 Japan; 5 Department of Palliative Care, Japan Red Cross Medical Center, 4-1-22 Hiroo, Shibuya-ku, Tokyo, 150-0012 Japan

**Keywords:** Cancer pain, Pain management index (PMI), Quality indicator, Pain interference, Palliative care

## Abstract

**Background:**

The Pain Management Index (PMI) is widely used in the assessment of pain management, and negative scores are traditionally considered to indicate inadequate pain management. However, it is not known whether negative PMI scores are always problematic.

**Methods:**

In this prospective observational study, we examined the data of 1156 patients with cancer and pain who were hospitalized in a cancer care hospital in Japan from July 2012 to January 2015 and compared the proportion of patients with PI across various PMI scores in this cohort. We further evaluated the predictive validity of PMI scores for PI using different cutoffs. This study aimed to examine the association between PMI scores and the proportion of patients whose pain interferes with their daily lives (i.e., pain interference [PI]).

**Results:**

We found that lower PMI scores were generally associated with a higher percentage of patients with PI. A smaller proportion of patients with PMI scores of − 1 (567/1550, 36.6%) reported PI compared with those with PMI scores of 0 (788/1505, 52.4%). The sensitivities of PMI scores < − 1 and < 0 for predicting PI were 0.16 and 0.37 and the corresponding specificities were 0.95 and 0.71, respectively.

**Conclusions:**

These findings suggest that PMI scores are inversely associated with the proportion of patients with PI. However, PMI scores of − 1 do not always indicate inadequate pain management; pain management should therefore be evaluated from multiple perspectives.

## Background

A substantial proportion of patients with cancer experience pain [1], which sometimes interferes with their daily lives and affects their mental health [2, 3]. As stated in the World Health Organization (WHO) guidelines on pain relief and palliative care, assessment of pain and providing appropriate treatment for it are important aspects of pain management [4]. Systematic evaluation is indispensable for ensuring appropriate pain management.

The Pain Management Index (PMI) is used worldwide to evaluate cancer pain management [5]. The PMI evaluates pain management by reflecting the balance between the class of the most potent analgesics used/prescribed and the patient’s reported pain intensity. Analgesic drugs and pain intensity are assigned scores of 0 to 3; subtracting the latter from the former provides the PMI. Thus, when the score for pain intensity is larger than the analgesic drug score, the PMI is negative; this has traditionally been considered to indicate inadequate pain management [6]. In a systematic review published in 2008, the prevalence of negative PMI scores among patients with cancer was reported as 43% worldwide, and higher in Asia [5]. A more recent report, published in 2014, showed slight improvement, the prevalence of negative PMI scores being 31.8% [7]. However, according to conventional criteria, this means that approximately one third of patients still do not receive pain medication that is proportional to their pain intensity [7].

Another indicator for evaluating pain management is pain interference [PI], defined as whether pain interferes with daily life. The Brief Pain Inventory (BPI), another popular instrument for assessing pain, assesses the intensity of pain and interference from pain in seven areas of daily life [8]. PMI and BPI scores are often used to assess patients’ pain status and the adequacy of analgesia [3]. The intensity of pain reflects only the pain, whereas the PI assesses the effectiveness of pain management by using measures like the PMI.

According to Deandrea et al., the process–outcome link is an important validity check for any performance measure [5]. However, the relationship between PMI and PI scores in patients with cancer has not been evaluated since the PMI was first developed in 1994, when Cleeland et al. compared PI scores between patients with negative PMI scores and those with scores of ≥zero [6]. However, a non-negative PMI value does not necessarily mean that the patient’s pain management is appropriate, because PMI only looks at the analgesic classes and the levels of pain. [9–11]. The further steps of analgesic choice and appropriate dosage are necessary to achieve appropriate pain management. In addition, negative PMI scores may not indicate inadequate pain management in patients without PI. This possibility was raised during the process of practice reviews of pain management in our institution. Several clinicians whose pain management practice was considered inadequate based on the PMI are opposed to the criteria, claiming that “inadequate pain management” is a false accusation because patients whose PMI scores were − 1 are not necessarily troubled by pain.

We performed this study to test these clinicians’ claims by analyzing in more detail the overall trend in PI scores across various PMI and PI scores. We aimed to assess the performance of the PMI as an indicator of the quality of pain management by relating the scores to PI, an outcome measure of pain management. We also expected that differences in prevalence of PI across PMI scores would provide insights on the how PMI scores should be used to assess quality of pain management.

## Methods

### Setting, participants, and data collection

Participants were recruited for this study from patients with cancer who were hospitalized at Aomori Prefectural Central Hospital, a general hospital in rural northern Japan, between July 2012 and January 2015. This hospital has approximately 700 beds and provides care to approximately 1200 new patients with cancer annually. All patients aged 20 years and older with a diagnosis of cancer who had cancer-related pain or had taken any analgesic medications in the past 24 h were invited to participate, provided their primary physicians assessed that they were capable of participating in the study. Participants were asked by staff nurses about pain and cancer-related PI as part of routine care during morning rounds every day. The patients’ reports on pain status during hospital days 1 to 7 were analyzed. Medications used were recorded by a staff nurse, who also documented the cause of pain (e.g., cancer- or treatment-related).

### Measures and definitions of variables

#### Pain and pain interference [PI]

Patients who had experienced cancer-related pain or taken any analgesic drugs in the past 24 h were eligible for the study. Given that patients with cancer can experience pain caused by various factors, including cancer itself, treatment, and coexisting illness [12], patients with pain other than cancer-related pain were excluded.

PI was defined as cancer pain that resulted in the patient feeling troubled or being incapable of doing something. Participants were asked a single question “Do you have trouble with pain or is there anything you cannot do because of pain?” They could respond “yes” or “no” [13].

#### Pain management index

Pain intensity can be determined at its strongest, as the average amount of pain in the past 24 h, or at rest and in motion. The strongest pain in the past 24 h was chosen as the basis for the PMI, in accordance with the original method of Cleeland et al. [6]. Pain intensity was reported on a numerical rating scale (NRS) from 0 to 10, with zero denoting no pain and 10 the worst pain imaginable. Information on prescribed analgesics (name, dose, frequency, and routes of administration) was collected from the medical record and patients’ responses to the staff nurses’ questions.

PMI scores were calculated by subtracting the pain score from the analgesics score as in the WHO pain relief ladder [6]. An NRS was used to ascertain the worst pain intensity and these scores used to create the following four categories of pain intensity: no pain (pain score 0; 0 on NRS); mild pain (pain score 1; 1–4 on NRS), moderate pain (pain score 2; 5 or 6 on NRS), and severe pain (7–10) [14]. An analgesic score based on the most potent analgesic prescribed to the patient at the time of the survey was assigned according to the WHO pain relief ladder as follows: 0, no analgesic medication; 1, non-opioid analgesics, such as non-steroidal anti-inflammatory drugs or acetaminophen; 2, weak opioids; and 3, strong opioids. Analgesics not classified in the WHO pain relief ladder were assigned an analgesic score of “0”.

### Data analysis

The proportion of patients with PI for each PMI score from − 3 to + 3, overall, and stratified by hospital day were calculated, after which the trend in proportion of patients with PI for each PMI score was assessed by using the Cochran–Armitage test. A *P* value of less than 0.05 was considered to indicate statistical significance. To further clarify the relationship between PMI scores and PI, the distribution of pain intensity experienced within each PMI score category was calculated. For the comparison of proportions of patients with the PI across categories, Pearson’s chi-square test was used. Because the ultimate target of pain management is to eliminate PI, the PMI can be viewed as a tool that tests “inadequate pain management” and thus assesses the process of achieving this target. Therefore, sensitivities and specificities of PMI < − 1 and PMI < 0 for predicting PI were computed. Although PMI < 0 is the conventional criterion for inadequate pain management, a PMI of − 1 is a looser criterion. The sensitivity of the PMI score was defined as the proportion of patients with PI who actually scored positive (i.e., PMI < − 1 or PMI < 0), and the specificity was defined as the proportion of patients without PI who scored negative (i.e., PMI > = − 1 or PMI > =0). All analyses were performed with Stata version 13.1 (StataCorp LP, College Station, TX, USA).

## Results

### Characteristics of study patients

**Fig. 1:**
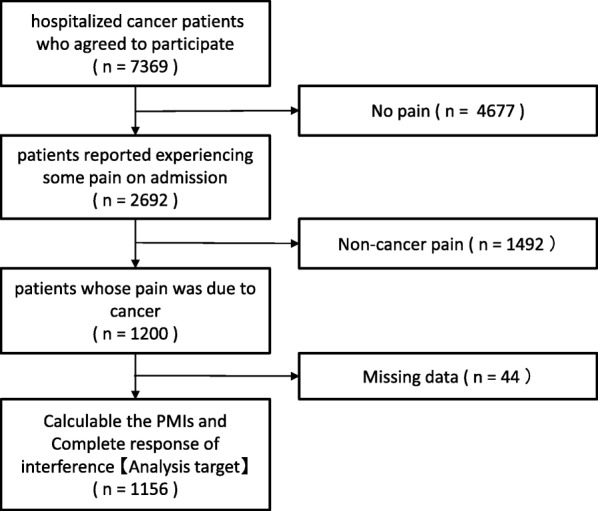
Flow diagram of the study

Of 7369 patients with cancer who agreed to be screened for pain as potential participants in the study, 2692 reported experiencing some pain on admission. Among the 1200 patients whose pain was attributable to cancer, 1156 reported pain on the NRS and were eligible for analysis. A total of 6732 responses about pain from these 1156 patients were analyzed (Fig. 1. On average, patients reported pain on the NRS 5.8 times). Table 1 presents these patients’ characteristics and condition on the first hospital day. Their mean age was 62.9 years (SD, 11.6 years) and 500 (43.3%) were women, 71.9% of them had a performance status of 0 or 1, 33.8% of them were not given prescriptions for analgesics, a little less than half of whom (41.2%) reported mild pain and 20.1% no pain. The average NRS value of patients with PI was 5.2, whereas that of patients without was 2.0 (*P* < 0.001).

**Table 1:** Relevant clinical characteristics (*n* = 1156)

Age — mean ± *SD*	62.9 ± 11.6
Female sex — no. (%)	500 (43.3)
Hospitalized Department no. (%)	
Surgery	227 (19.6)
Gastroenterology	400 (34.6)
Respiratory Meds	213 (18.4)
Hematology	87 (7.5)
ENT	71 (6.1)
Urology	58 (5.0)
Gynecology	86 (7.4)
Other	14 (1.4)
Analgesics^a^ — no. (%)	
None	391 (33.8)
Non opioids	321 (27.8)
Mild opioids	47 (4.1)
Strong opioids	397 (34.3)
Performance Status — no. (%)	
0	456 (39.5)
1	374 (32.4)
2	159 (13.8)
3	118 (10.2)
4	49 (4.2)
Treatment history — no. (%)	
Surgery	463 (40.1)
Chemotherapy	671 (58.0)
Radiation therapy	244 (21.1)
NRS — no. (%)	
0 (No pain)	232 (20.1)
1–4 (Mild pain)	476 (41.2)
5–6 (Moderate pain)	191 (16.5)
7–10 (Severe pain)	257 (22.2)

^a^Classification according to the WHO’s pain relief ladder. Non-opioids included non-steroidal anti-inflammatory drugs or acetaminophen. Analgesics not classified in the WHO’s pain relief ladder, such as adjuvant analgesics, were assigned to “none” in this study

*NRS* numerical rating scale

### Relationship between PMI scores and PI

**Fig. 2:**
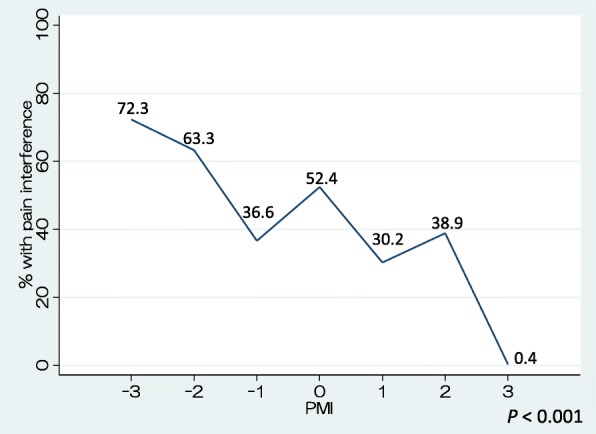
Relationship between PMI scores and pain interference (sum of all days). The percentages are of patients with PI at each level of PMI score. *P* value: Cochrane-Armitage test for trend was performed

**Fig. 3:**
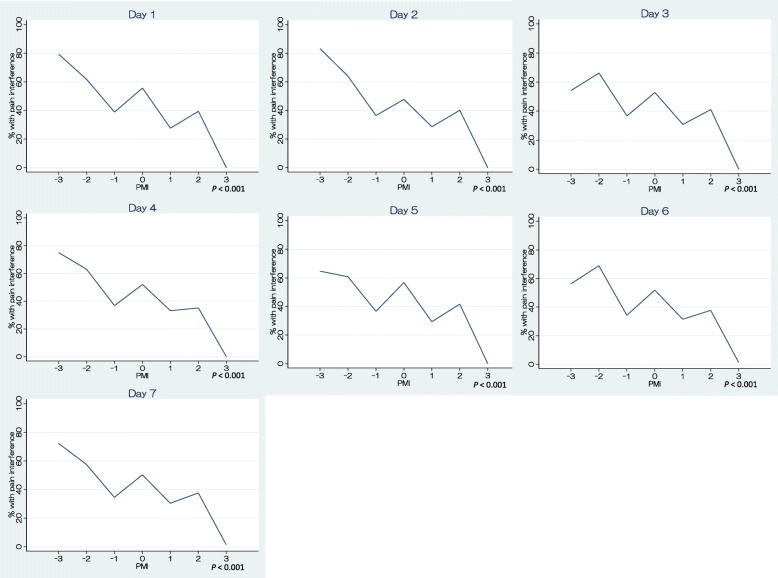
Relationship between PMI scores and pain interference (each PMI level stratified by hospital day). *P* value: Cochrane-Armitage test for trend was performed

Figure 2 shows the proportions of patients who reported PI according to PMI score. Overall, there was a significant trend toward lower PMI being associated with a higher proportion of patients with PI (*P* < 0.001). A large proportion of patients with PMI scores of − 2 or − 3 reported PI, whereas this proportion was much smaller among those with PMI scores of − 1, approximating the proportions of those with positive PMI scores of 1 or 2; no patients with positive PMI scores of 3 reported PI. The proportion of patients with PI was lower for those with PMI scores of − 1 than for those with PMI scores of 0 (*P* < 0.001). Figure 3 shows the same analyses as Fig. 2, stratified by hospital day (1–7); the graphs are of similar shape as that in Fig. 2. Except for on days 3 and 6, the proportion of patients reporting PI was greater in those with PMI scores of − 3 than in those with PMI scores of − 2; on days 3 and 6 the findings for these PMI scores were reversed.

### Distribution of pain intensity and PI according to category of PMI scores

**Fig. 4:**
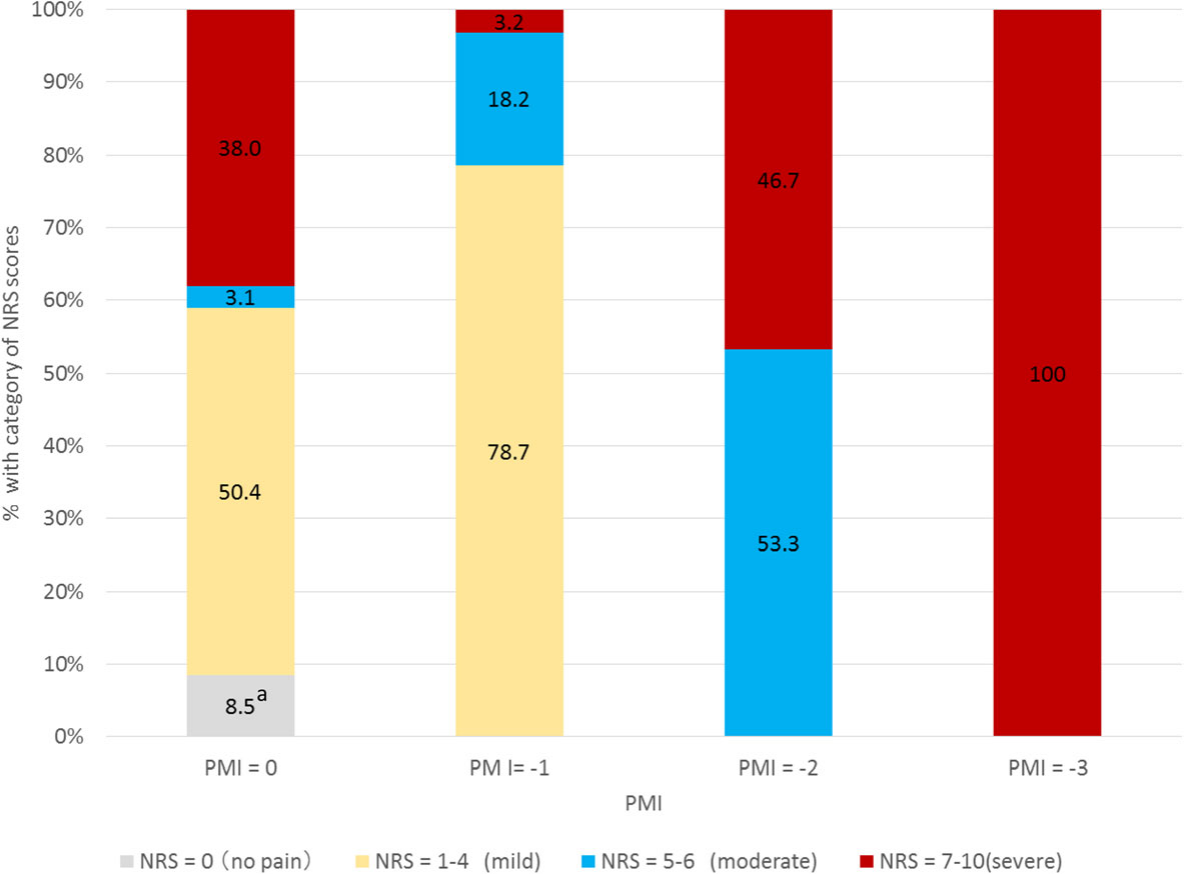
Distribution of patients in categories of PMI scores and pain intensity. “a”, percentage of patients whose pain was relieved by adjuvant analgesics or by analgesics that are not classified in the WHO pain relief ladder. NRS, Numeric Rating Scale. NRS scores range from 0 to 10, lower scores indicating less severe pain, scores grouped into four categories

Figure 4 shows the distribution of pain intensity in patients with PMI scores ≤0. Most (78.7%) patients with PMI scores of − 1 had mild pain; 18.2% and 3.2% and had moderate and severe pain, respectively. However, 38.0% of patients with PMI scores of 0 had severe pain (38.0%). Table 2 shows the proportion of patients with PI according to category of PMI score or pain intensity group. At the same levels of PMI scores, there was a larger proportion of patients with greater pain intensity reported PI, indicating correct estimation of PI.

**Table 2:** Proportion of patients experiencing PI according to category of PMI scores and pain intensity

pain intensity (NRS)/PMI	PMI = 0	PMI = −1	PMI = −2	PMI = −3
NRS = 0 (no pain)	0 /128 (0%)			
NRS = 1–4 (mild pain)	278/758 (36.7%)	339/1219 (27.8 %)		
NRS = 5–6 (moderate pain)	30 /47 (63.8%)	191/282 (67.7%)	122/234 (52.1%)	
NRS = 7–10 (severe pain)	480/572 (83.9%)	37/49 (75.5%)	156/205 (76.1%)	136/188 (72.3%)
Total	788/1505 (52.4%)	567/1550 (36.6%)	278/439 (63.3%)	136/188 (72.3%)

*Note*: The numbers of each cell are shown, the denominator is the number of patients who experienced cancer pain, and the numerator is the number of patients who had pain interference due to cancer. *NRS* numerical rating scale. The numbers in this table are cumulative number because it contains Days 1–7 including patients who were discharged in fewer than 7 days

The sensitivities of PMI scores < − 1, < 0 and < 1 for predicting PI were 0.16, 0.37, and 0.67, respectively; the corresponding specificities being 0.95, 0.71, and 0.53, respectively.

## Discussion

Negative PMI scores are widely accepted as indicating inadequate management of pain, whereas non-negative (0 to 3) scores are thought to denote acceptable pain management [5, 7, 11, 15, 16]. Indeed, we found a trend toward the proportion of PI decreasing as PMI scores increased, and that a large proportion of patients with PMI scores of − 2 or − 3 reported PI. However, we found that a larger proportion of patients in the group with PMI scores of 0 reported PI than in the group with PMI scores of − 1. This finding indicates that patients with a PMI score of − 1, traditionally considered as “treated inadequately”, were experiencing less interference with daily living than those with PMI scores of 0, usually considered as receiving “acceptable pain management”.

This finding suggests caution must be exercised when evaluating pain management using the PMI. A PMI score of − 1 should be considered as ambiguous, rather than denoting inadequate pain management, and treated differently according to the purpose of the evaluation. When the aim is to identify definitively inadequate care, PMI scores of − 2 and − 3 should be considered, and not scores of − 1. However, when the aim is to broadly capture potentially deficient care for further evaluation, patients with scores of 0 and − 1 should be included. In previous studies that used the PMI to evaluate the quality of pain management, all negative PMI scores were considered to denote inadequate pain management [5, 7, 11, 15, 16]. Thus, the criteria may need to be re-assessed according to the purpose of administering the PMI. Additionally, to take the other perspective, non-negative PMI scores do not necessarily indicate acceptable care: a substantial proportion of patients with PMI scores of 0–2 reported PI. Although we do not have information on the severity of that PI, these patients warrant attention [9–11].

The explanation for fewer persons with PMI scores of − 1 having PI than those with PMI of 0 may relate to the difference in distribution of pain intensity experienced by these patients. Most patients with PMI scores of − 1 had untreated mild pain, whereas those with PMI scores of 0 had a more balanced distribution of mild and severe pain. A possible reason for this finding is that patients with some mild pain decline pain medications because they have no PI, or because of cost or other reasons; this may be true of the group of patients with PMI scores of − 1 in our study. Scenarios such as patients with mild pain receiving no analgesics versus patients with moderate or severe pain taking appropriate classes of analgesics require more attention when exploring how to improve pain management. Assessment of quality of pain management by the PMI at best reflects only one aspect of the multidimensional factors involved in pain and pain management.

Our study has several limitations. First, it was conducted in a single facility in rural Japan, is a cancer care hospital that plays a central role in cancer diagnosis and treatment in a prefecture of Japan. Most of the patients with cancer were in the early stages and receiving active anti-tumor treatment. Thus, our results may not be generalizable to other settings, such as hospitals in large cities that have a higher proportion of patients receiving end-of -life care. However, even in different types of settings, we consider that not all negative PMI scores indicate inadequate pain management. Evaluation needs to be more comprehensive and consider various factors. Second, we used PI as a dichotomous variable, that is, we did not take into account the extent of PI. PI may be more severe in patients with large negative PMI scores than those with other scores. Assessing only the proportion of patients reporting PI may have resulted in missing some important aspects of the problems. Additionally, the simplicity of the single question used to assess PI meant that it did not go through as rigorous a validation process as when developing the measurement tool. It is possible that some patients misunderstood the PI question. However, the mean value of the NRS of pain in the no PI patients was substantially lower (mean 2.0) than that of patients with PI (5.4). Third, PI is a subjective measure and can therefore be affected by patients’ personal perceptions of pain, expectations of their own function, their ability to coping with their pain, and lifestyles. An example of this is that bedridden patients may have low expectations concerning function and may therefore report no interference by pain. Additionally, PI has the limitation that patients sometimes have difficulty in distinguishing interference by pain itself from limitation by other factors, such as their general physical condition. Furthermore, similar to other outcome measures (e.g., survival), PI is an inclusive measure of care, and is sometimes influenced by factors other than care. Finally, in the present study PI was assessed from the patient’s response to a single question. This could account for slight differences in results between this study and other studies that assessed PI by other means.

## Conclusions

Lower PMI scores predict a large proportion of patients who experience interference with their daily lives by pain. However, negative PMI scores do not necessarily indicate that pain is interfering with a patient’s daily life, and non-negative PMI scores do not necessarily indicate adequate pain management. The PMI is a useful tool for quickly assessing pain management. However, it captures only one aspect of pain management, namely the balance of pain intensity and analgesics used. The PMI misses other important factors that may affect patients’ wellbeing, such as dosage of analgesics and response to breakthrough pain. Our findings also indicate that judging the quality of pain management solely on PMI scores is an oversimplification of a complex issue. The PMI is effective in promoting assessment of care in broad settings; however, detailed interpretation by other means would facilitate further improvement in assessment of pain management.

## Abbreviations

BPI: The Brief Pain Inventory
NRS: A Numerical Rating Scale
PI: Pain Interference
PMI: The Pain Management Index
